# Complete seizure freedom following minimally invasive laser callosotomy in a patient with lissencephaly type 1: Challenging the traditional paradigm

**DOI:** 10.1002/epd2.70307

**Published:** 2026-06-22

**Authors:** Agilda Dema, Archie Baskaran, Joshua Chang, Douglas Nordli, Peter Warnke

**Affiliations:** ^1^ Department of Pediatric Neurology University of Chicago Chicago Illinois USA; ^2^ Department of Neurology University of Chicago Chicago Illinois USA; ^3^ Department of Neurological Surgery University of Chicago Chicago Illinois USA

**Keywords:** callosotomy, laser interstitial thermal therapy (LITT), Lennox‐Gastaut syndrome, lissencephaly type 1, refractory epilepsy, syndrome: Lennox‐Gastaut Syndrome, etiology: lissencephaly, phenomenology: generalized, tonic seizure, localization: generalized, multifocal

Lissencephaly type 1 is a severe cortical malformation characterized by a smooth cerebral surface, abnormal cortical layering, profound developmental delay, and highly refractory epilepsy.^1^ While resective epilepsy surgery is often limited due to diffuse cortical involvement and poor seizure localization, palliative interventions such as corpus callosotomy and neuromodulation are commonly performed.^2^ Advances in minimally invasive techniques, including MRI‐guided laser interstitial thermal therapy (LITT), have expanded surgical options for drug‐resistant epilepsy.^3^

We report a 19‐year‐old female with lissencephaly type 1, static profound global developmental delay, severe intellectual disability, and drug‐resistant epilepsy consistent with Lennox–Gastaut syndrome (LGS), who achieved complete seizure freedom following MRI‐guided LITT complete corpus callosotomy.

Seizure onset occurred at 3 months of age, initially presenting with behavioral arrest and eyelid myoclonia. Neuroimaging confirmed lissencephaly. Despite multiple antiseizure medications during infancy, she experienced more than 100 seizures daily. Over time, her epilepsy evolved, and by adolescence, seizures were characterized by generalized tonic–clonic seizures. She also exhibited tonic seizures and atonic drop attacks. Seizures were previously triggered by photic and auditory stimuli and demonstrated a catamenial pattern. At baseline, she was nonverbal with severe intellectual disability and fully dependent for all activities of daily living (Video 1).

**VIDEO 1:** MRI‐guided laser interstitial thermal therapy (LITT) corpus callosotomy. This video demonstrates intraoperative MRI thermometry during LITT‐assisted complete callosotomy. Real‐time, color‐coded thermal maps guide controlled ablation of the corpus callosum, ensuring effective disconnection while preserving adjacent cortex. Left panel: Stereotactic trajectory planning and laser probe placement via an interhemispheric approach targeting the corpus callosum. Right panel: Real‐time MRI thermography‐guided ablation showing progressive thermal lesioning and callosal fiber disconnection with continuous monitoring. Video content can be viewed at https://onlinelibrary.wiley.com/doi/10.1002/epd2.70307.

Her epilepsy remained highly refractory despite treatment with valproate, clobazam, levetiracetam, topiramate, and lamotrigine. A vagus nerve stimulator implanted at age 8 remained active at the time of surgery but provided minimal clinical benefit despite optimization of stimulation parameters. A ketogenic diet initiated at age 16 initially reduced seizure frequency to 5–8 seizures daily but did not achieve adequate control. Prior to callosotomy, seizure burden escalated to frequent axial and rhizomelic tonic seizures during sleep, clustering up to 35–45 episodes per hour and severely disrupting her sleep–wake cycle. Seizures significantly impaired quality of life, contributing to weight loss, recurrent infections, and frequent hospitalizations (Figure 1).

**FIGURE 1:**
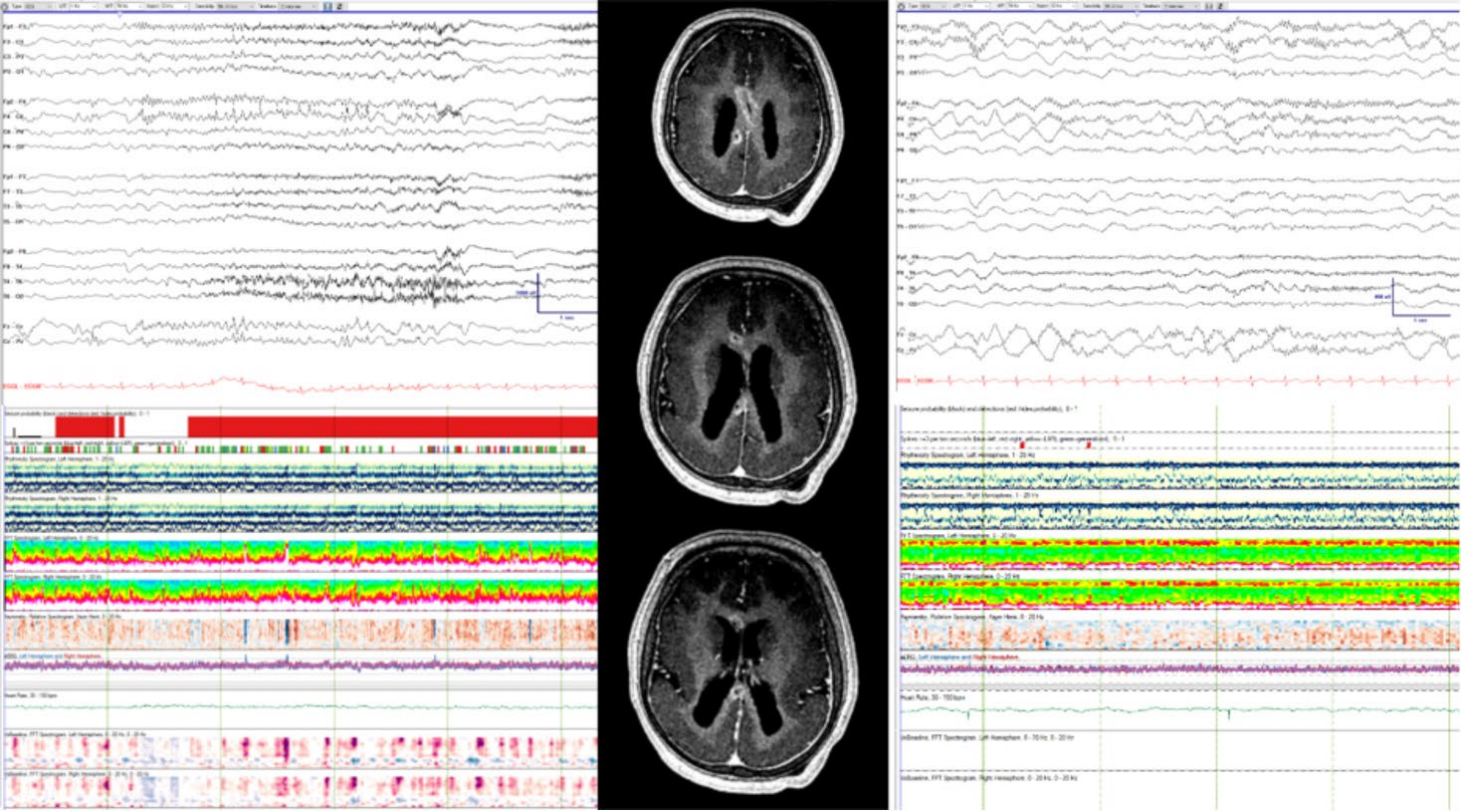
The middle panel shows an intraoperative axial T1‐weighted MRI obtained during MRI‐guided laser interstitial thermal therapy (LITT) ablation. EEG recordings before LITT (left) and at 3 months following LITT corpus callosotomy (right) demonstrate a transition from disorganized background activity with frequent multifocal discharges and high seizure burden to reduced epileptiform activity and sustained seizure freedom.

Preoperative evaluation demonstrated generalized epileptiform activity on electroencephalography consistent with LGS and imaging findings consistent with lissencephaly type 1. Given persistent drug‐resistant generalized seizures and drop attacks, she underwent minimally invasive complete corpus callosotomy using LITT at 18 years of age.

Postoperatively, seizure frequency decreased progressively over several weeks, with complete seizure cessation achieved by approximately 3 months. At last follow‐up, she has remained seizure‐free for 10 months postoperatively, corresponding to Engel Class I (currently most consistent with Class I‐A). No new neurological deficits were observed. In parallel, she demonstrated normalization of her sleep–wake cycle, improved alertness, and improved overall health. Previously identified seizure triggers were no longer observed, and she has not required hospitalization since surgery.

The delayed onset of seizure freedom suggests that therapeutic benefit may extend beyond immediate interruption of interhemispheric propagation.^4^ Proposed mechanisms include disruption of pathological interhemispheric synchrony, progressive desynchronization of epileptogenic networks, and Wallerian degeneration of transcallosal fibers leading to gradual attenuation of epileptogenic connectivity.^5^, ^6^ These findings further support the concept that disconnection procedures may induce evolving network‐level reorganization.

Corpus callosotomy is a well‐established palliative treatment for LGS, particularly for tonic seizures and drop attacks.^2^, ^7^ Prior studies of open callosotomy in LGS have demonstrated seizure reduction in a majority of patients, although complete seizure freedom remains uncommon, particularly in the setting of diffuse cortical malformations such as lissencephaly.^8^ Compared with open approaches, LITT offers reduced morbidity, shorter hospitalization, faster recovery, and precise MRI‐guided targeting, although long‐term comparative data remain limited.^3^, ^9^

Notably, reports of complete seizure freedom following corpus callosotomy in patients with lissencephaly are exceedingly rare, particularly with a minimally invasive LITT‐based approach.^10^ This case demonstrates that complete seizure freedom may be achievable following disconnection surgery in selected patients with lissencephaly type 1. However, the relatively short follow‐up duration limits conclusions regarding long‐term seizure freedom and durability of response. While limited to a single case, these findings support reconsideration of surgical options in selected patients with diffuse cortical malformations and drug‐resistant epilepsy.

## FUNDING INFORMATION

The authors received no financial support for the research, authorship, and/or publication of this article.

## CONFLICT OF INTEREST STATEMENT

The authors declare no conflict of interest.

## INFORMED CONSENT

Written informed consent was obtained from the patient's legal guardians for inclusion in this report. All identifying details have been omitted or anonymized to protect patient confidentiality.

**Test Yourself**

1. Which neuropathologic feature is most characteristic of lissencephaly type 1?
  - Focal cortical dysplasia limited to one lobe.
  - Polymicrogyria with excessive cortical folding.
  - Smooth cerebral surface with abnormal cortical layering.
  - Periventricular nodular heterotopia.
2. Which clinical scenario most appropriately supports consideration of corpus callosotomy in drug‐resistant epilepsy?
  - Seizures arising from a single, well‐localized temporal lobe focus.
  - Frequent generalized seizures with rapid bilateral synchronization.
  - MRI‐visible focal cortical dysplasia in non‐eloquent cortex.
  - Focal seizures with consistent unilateral EEG onset and preserved consciousness.
3. Which mechanism may contribute to delayed seizure improvement after callosotomy?
  - Wallerian degeneration of transcallosal fibers.
  - Increased cortical plasticity due to edema.
  - Immediate disruption of focal epileptogenic cortex.
  - Enhanced vagal tone.

*Answers may be found in the* Supporting Information.

## Supporting information


Data S1.

## Data Availability

The data that support the findings of this study are available from the corresponding author upon reasonable request.
