# Supplementary material for: Complete seizure freedom following minimally invasive laser callosotomy in a patient with lissencephaly type 1: Challenging the traditional paradigm
Source: Epileptic Disord. 2026 Jun 22;28(5):1705–8. doi: 10.1002/epd2.70307 (PMC13695473; doi:10.1002/epd2.70307)
Supplement: Supplementary file 1 [file EPD2-28-1705-s001.docx]

**Test Yourself Answers**

**1. Correct answer: C**
Lissencephaly type 1 results from impaired neuronal migration, leading to a smooth cortex and abnormal lamination.

**2. Correct answer: B**
Corpus callosotomy is most effective in generalized epilepsies characterized by rapid interhemispheric synchronization, such as tonic and atonic seizures.

**3. Correct answer: A**
Wallerian degeneration of transected commissural fibers occurs over weeks and may progressively reduce pathological interhemispheric connectivity, leading to delayed seizure improvement.
